# Genomic reconstruction of Bacillus anthracis from complex environmental samples enables high-throughput identification and lineage assignment in Pakistan

**DOI:** 10.1099/mgen.0.001422

**Published:** 2025-06-23

**Authors:** Justin C. Podowski, Sara Forrester, Tahir Yaqub, Amin Aqel, Mohammad Abu Lubad, Nadia Mukhtar, Muhammad Waqar Aziz, Nageen Sardar, Hassaan Bin Aslam, Hamda Pervaiz, Alan J. Wolfe, Daniel S. Schabacker

**Affiliations:** 1 Data Science and Learning Division, Argonne National Laboratory, Lemont, Illinois, USA; 2 Biosciences Division, Argonne National Laboratory, Lemont, Illinois, USA; 3 Institute of Microbiology, University of Veterinary and Animal Sciences, Lahore 54000, Pakistan; 4 Faculty of Medicine, Mu’tah University, Al-Karak 61710, Jordan; 5 Department of Microbiology, The Islamia University of Bahawalpur, Bahawalpur 63100, Pakistan; 6 Department of Microbiology, University of Jhang, Jhang 35200, Pakistan; 7 Department of Microbiology and Immunology, Loyola University Chicago, Maywood, Illinois, USA

**Keywords:** A.Br.047 Vollum, *Bacillus anthracis*, cgMLST, genomics, metagenomics, Pakistan

## Abstract

*Bacillus anthracis*, the causative agent of anthrax, is a highly virulent zoonotic pathogen primarily affecting domesticated and wild herbivores. Human exposure to *B. anthracis* is primarily through contact with infected animals or contaminated animal products. In Pakistan, where livestock vaccines are largely unavailable and infected carcasses are often disposed of improperly, the risk to humans, wildlife and livestock is significant. Currently, the diagnosis of anthrax infections and outbreak tracing necessitates the isolation and culturing of *B. anthracis*, a process that requires BSL-3 facilities. In this study, we show that positive identification, genome reconstruction and lineage assignment can be accomplished using bioinformatic analysis of DNA extracted directly from environmental samples that would otherwise provide the starting material for isolation and culturing. This approach does not require laboratory target enrichment as is necessary for other pathogens, due in part to the extremely high bacterial load in the bloodstream in the deceased animals. Using these methods, we greatly expand the knowledge of endemic *B. anthracis* in Pakistan. We provide the first reference *B. anthracis* genomes from Pakistan since the 1970s and identify A.Br.014 Aust94 as a minor circulating sublineage alongside the dominant A.Br.047 Vollum. Future work will focus on the limits of detection and will determine if this bioinformatic method can be expanded more broadly for *B. anthracis* or other pathogens to replace typical culture-based methods.

**Impact Statement**

Here, we present data collected from anthrax outbreaks in livestock in Pakistan between 2019 and 2021. These data include genomes from 10 isolates of *Bacillus anthracis* along with 31 complex environmental samples collected from around infected livestock. We demonstrate that shotgun metagenomic sequencing methods can be leveraged to reconstruct *B. anthracis*, and those genomes can be used to provide core genome MLST (cgMLST) data vital for outbreak tracing. As a result, this work suggests that the use of shotgun metagenomic sequencing of complex environmental or clinical sequencing could reduce the need to isolate *B. anthracis* and could allow *B. anthracis* outbreak tracing where appropriate BSL facilities are not available.

## Data Summary

Raw reads and assemblies for isolate genomes are available in BioProject PRJNA1159740. Raw reads for complex samples are available in BioProject PRJNA1159740. Documentation of code involved in genome assembly, as well as complex sample analysis, is available at https://github.com/jpod1010/pathogencomplexsamples/blob/main/pipeline_code.

## Introduction

*Bacillus anthracis* is a Gram-positive bacillus that causes anthrax, a high-mortality infection of humans and many wild and domesticated mammals. Soil is the natural reservoir for *B. anthracis*, and as a result, infection in wild and domesticated animals occurs largely through grazing. Infections in humans occur through contact with animal carcasses, meat and other animal products derived from those animals. As a result, major factors determining the rates of human infections are endemicity of *B. anthracis* in the soil and the degree of interaction between humans and infected animals or animal products. Whilst *B. anthracis* is present on all continents except Antarctica [1], the continents with the greatest risk to humans are Africa, Eurasia and North America [2]. Risks in Pakistan are particularly high due to low rates of livestock vaccination [2,3] and regular interaction with livestock due to large rural populations [4], including meat scavenging from carcasses [5]. As a result, *B. anthracis* is found routinely in soils inside of villages [6], suggesting the potential for non-zoonotic infection of humans directly from soil. A more complete understanding of the prevailing lineages of *B. anthracis* in Pakistan is vital to successful outbreak tracing, but very few sequenced isolates exist [7,8], limiting these efforts.

Detection of *B. anthracis* in a clinical or environmental sample can be accomplished through amplification of key virulence genes and/or specific chromosomal targets [9,10]. However, this does not allow for the phylogenetic inference of lineages, which is necessary for outbreak tracing and usually requires isolation of *B. anthracis* through culturing*,* DNA extraction and either variable number tandem repeat analysis [1] or bioinformatic analysis of whole-genome sequence data [7]. The limited availability of BSL-3 facilities necessary to culture *B. anthracis* in many countries most threatened by *B. anthracis* limits outbreak tracing [11]. Culture-independent analysis of other pathogens from clinical samples [12] suggests that environmental samples could potentially be leveraged to identify and reconstruct *B. anthracis* genomes.

Compared to other pathogens, the population structure and infection dynamics of *B. anthracis* present both challenges and opportunities to culture-independent analysis. High similarity between *B. anthracis* and related *Bacillus cereus* group organisms complicates the use of small-subunit-based marker approaches [13,14], and the cryptic presence of infectivity plasmids in *B. cereus* group organisms complicates the use of these infectivity genes [15]. However, the extremely high abundance of *B. anthracis* in the blood of infected animals [16] means that amplification or target enrichment may not be necessary, as it is for other pathogens [17].

Here, we show that *B. anthracis* complete genomes can be reconstructed from blood-stained soil samples collected near animals that had died from anthrax infections and that these recovered genomes can be used to identify lineages necessary for outbreak tracing. Overall, we demonstrate the feasibility of culture-independent methods to surveil *B. anthracis*, whilst broadly expanding knowledge of endemic *B. anthracis* in Pakistan.

## Methods

### Sample collection

Samples in this study were selected from a subset of those collected for a previous anthrax surveillance programme. Between March 2019 and July 2021, a total of 570 soil samples were collected as described previously [5]. In brief, we defined an outbreak as a single dead livestock animal that exhibited signs consistent with anthrax (blood oozing from carcass’s orifices, bloating and absence of rigour mortis). Blood-stained soil samples were collected near the mouth or anus of the animal. Several samples per outbreak were collected, varying between one and six samples. Blood-stained soil samples were processed for the isolation of *B. anthracis* spores using the previously described GABRI method [18]. For *B. anthracis* isolates, blood-stained soil was used to generate cultures using selective media (PLET Agar) and differential media (Blood Agar) in parallel. Of the 570 soil samples collected, 65 were found to be positive for *B. anthracis*.

After the conclusion of the previous anthrax surveillance programme [5], 39 soil samples with sufficient remaining material for DNA sequencing were identified. Ten of these 39 samples also had paired *B. anthracis* isolates. These samples with sufficient remaining material that were Gram-positive, containing green-coloured spores in elongated chains, and were PCR-positive for all five of *pag* (PA), *cya* (EF), *lef* (LF), *capBCA* (CAP) and Ba813 (chromosomal) [19] were leveraged for this work. DNA was extracted from all sample types using a QIAamp DNA Mini Kit (Qiagen, Germantown, MD). All DNA samples were subjected to Health and Human Services (HHS) regulations to ensure that no viable *B. anthracis* was present in DNA extracts (HHS validation of *Bacillus anthraics* inactivation, HHS-0920-2018-F-2274), as well as USDA regulations to ensure that no viable foot-and-mouth disease virus was present. DNA samples were then shipped to the USA for sequencing.

### DNA sequencing

For all samples, library preparation was carried out using the Illumina DNA Prep tagmentation kit (Illumina, San Diego, CA). Isolated genome samples were sequenced on an Illumina NextSeq 2000 at 400 Mbp at the Microbial Genome Sequencing Center LLC (Pittsburgh, PA). Complex samples were sequenced on an Illumina NextSeq 2000 at 2 Gbp. For selected complex samples ZBM8S2_S1, ZBM8S3_S2, ZBM8S5_S3, ZBM8S6_S4, ZBM11S2_S5, ZBM11S4_S6 and ZBM11S7_S7, library preparation and sequencing were instead carried out at Argonne National Laboratory Environmental Sample Preparation and Sequencing Facility. Library preparation was carried out using the PrepX^™^ DNA Library Kit (Takara Bio USA, San Jose, CA), and samples were sequenced on an Illumina NextSeq 2000 at up to 20 Gbp per sample. Of the 39 soil samples with sufficient material, libraries were successfully prepared for 31 samples, along with all ten isolate DNA samples.

### Bioinformatic analysis

Unless otherwise stated, defaults were used for bioinformatic software. Raw reads from isolate *B. anthracis* genomic data were quality-controlled using BBTools v39.06 [20]. BBduk with ktrim=r k=23 mink=11 hdist=1 tpe tbo was used to remove adapters, removehuman.sh was used to remove human reads, BBduk trimq=30 qtrim=r was used to quality control and BBnorm target=100 mindepth=2 was used to normalize read depth. SPAdes v3.15.5 [21] was used to assemble these reads into contigs with the –isolate flag. Stats.sh from BBTools was used to assess contig size.

Raw reads from complex samples had adapters removed, human reads removed and sequences quality-controlled using the same methods as for isolate genome samples, including digital normalization to a target of 100 and a min depth of 2. However, digitally normalized reads were used for assembly and not for quantification of coverage. To assess the percentage of a short read sample that was definitively *B. anthracis*, KrakenUniq [22] was used to compare sequences against the precompiled MicrobialDB, for both whole-genome samples and complex samples. Multiple distinct methods were then used to test the efficiency of bioinformatic extraction of *B. anthracis* DNA sequences from metagenomic sequence data derived from complex samples.

Multiple bioinformatic methods (untargeted, semi-targeted, targeted and highly targeted) were tested to determine how *B. anthracis* genomes could be most efficiently recovered. Untargeted and semi-targeted methods that used metagenomic binning approaches were attractive because they allow for isolation without a reference, which could potentially enforce results similar to the reference used. Whereas targeted and highly targeted methods have the potential downside of enforcing similar results to the reference used, they are much quicker and require fewer computational resources.

For method 1 (untargeted), reads were normalized using BBnorm target=100 mindepth=2 and then assembled using MEGAHIT v1.2.9 [23] with –presets meta-sensitive. Metagenomic binning was carried out in metaWRAP v1.3.2 [24]. Bowtie2 v2.3.5.1 [25] was used to generate coverage for contigs, and Metabat2 v 2.12.1 [26], MaxBin2 v2.2.6 [27] and CONCOCT v1.0.0 [28] were all used to identify bins. MetaWRAP bin_refinement was used with -c 75×5 to identify high-completion bins using CheckM v1.0.12 [29]. As a result, a high-quality genome was defined as being more than 75% complete and less than 5% contaminated.

For method 2 (semi-targeted), reads were normalized and assembled as in method 1. Assemblies were then submitted to the Bacterial and Viral Bioinformatics Resource Center (BV-BRC) [30] where metagenomic binning was carried out [31] using what we deemed ‘semi-targeted’ binning. We refer to this binning as ‘semi-targeted’ because the binning method leverages several steps, such as blast similarity to a database of core genes, that target known organisms with genomes present in the BV-BRC database. Whilst this does not explicitly target user-specified genomes, it necessarily is biassed towards the recovery of known genomes, in contrast to coverage and composition-based metagenomic binning employed in method 1, which does not carry that bias. A high-quality genome was defined here by the standards used in BV-BRC [31].

For method 3 (targeted), a Bowtie2 index was created using the *B. anthracis* ‘Ames Ancestor’ strain (GCF_000008445.1), and reads were mapped against that index using Bowtie2 --sensitive --no-unal. Read pairs where both reads were mapped were extracted from the resultant samfile, and SPAdes was used to assemble using –isolate. Here, any assembly which was successfully completed was defined as a genome and subjected to further analysis.

For method 4 (highly targeted), analysis was identical to method 3, except that --trusted-contigs to the GCF_000008445.1 assembly fasta was used. This was done to enforce reference-based assembly. As above, any assembly which was successfully completed was defined as a genome and subjected to further analysis.

GTDB-Tk v2.1.1 [32] with database release 214 was then used to identify the taxonomy of genomes from all methods to identify *B. anthracis*. Once high-quality *B. anthracis* genomes were identified from either isolates or complex samples, CanSNPer v1.0.10 was used to assign sublineage, and PubMLST [33] was used to assign core genome MLST (cgMLST) profiles specific to *B. anthracis* [7] and compare genomes from this study to others in the PubMLST database.

ArcGIS Pro v3.2 was used to generate maps depicting the geographic distribution of sampling and cgMLST assignments.

All work was carried out on a dual-socket compute node with 2 Intel(R) Xeon(R) Gold 6128 CPU @ 3.40 GHz processors for a total of 12 cores and 24 threads available, and a total of 500 GB of RAM.

## Results

From previous anthrax surveillance efforts [5], samples with sufficient material were selected from outbreaks across the districts of Zhob, Bajour and Bahawalnagar in Pakistan. Genomic DNA from isolates from five outbreaks was acquired, representing ten isolates in total. Metagenomic DNA from the complex sample (blood-stained soil) that generated each of these ten isolates was also available and paired with those ten isolate DNA samples. An additional 21 complex samples from an additional eight outbreaks were also selected to expand surveillance of sublineages beyond outbreaks where isolates could be generated. In total, this represented samples from 13 outbreaks.

### Isolate genomes

High-quality isolate genomes were successfully obtained from all ten isolate samples. For all ten samples, KrakenUniq identified 99% of the reads in each sample as *B. cereus* group and identified at least 78%–81% of the reads specifically as *B. anthracis*. Assembled genome sizes ranged from 5.5 to 6.5 Mb, and, on average, 93% of those genomes were contained within contigs larger than 50 kb (Table 1).

**Table 1.** Description of the ten genomes from *B. anthracis* isolates from Pakistan. KrakenUniq was used to assign % of *B. anthracis* reads against the MicrobialDB database. cgMLST, which is a standardized nomenclature for closely related strains based on thousands of SNPs, was assigned using PubMLST [33]

Sample name	cgMLST	% reads of *B. anthracis*	Isolate genome size	% genome in scaffolds>50 kb
ZBMS4	A.Br.047	79	5.470 Mb	96
ZBMS6	A.Br.047	79	5.476 Mb	96
ZBM2S3	A.Br.047	78	5.471 Mb	96
ZBM2S5	A.Br.047	78	5.501 Mb	95
ZBM11S7	A.Br.047	78	5.473 Mb	96
ZBM11S8	A.Br.014	79	5.727 Mb	92
BW7S4	A.Br.014	79	6.490 Mb	81
BW7S5	A.Br.014	79	5.468 Mb	96
BW10S3	A.Br.014	80	6.251 Mb	82
BW10S5	A.Br.014	80	5.472 Mb	95

Five isolate genomes were assigned to the A.Br.007 Vollum clade, and five were assigned to the A.Br.003 Aust94 clade, using CanSNPer [1,34]. Concordantly, the five assigned A.Br.007 were identified more specifically as A.Br.047 by cgMLST [7,33], whilst those assigned A.Br.003 were more specifically assigned A.Br.014. Using the assigned cgMLST profiles, PubMLST [33] was used to identify the closest existing isolates. The closest existing isolate for the A.Br.047 genomes was SK-102 (GCF_000832565.1), which was isolated from wool originating in Pakistan in 1976. The closest isolate for all A.Br.014 genomes was A0656 (GCA_029700625.1), which was isolated from soil in China in 1982.

### Genome reconstruction

Thirty-one DNA samples derived from blood-stained soil were sequenced. For these 31 samples, the percentage of positively identified *B. anthracis* sequences ranged from 1.65% to 81%, with an average of 46% across all 31 samples. Of these 31 samples, two samples did not produce a reconstructed *B. anthracis* genome using any method. Both of these samples produced reconstructed genomes identified as *B. cereus* using untargeted and semi-targeted methods.

Across four bioinformatic methods – untargeted, semi-targeted, targeted and highly-targeted – we attempted to reconstruct *B. anthracis* genomes from which lineage information could be assigned (Table 2). Untargeted methods produced a *B. anthracis* genome in 20 samples, semi-targeted in 23 samples, targeted in 27 samples and highly targeted in 31 samples (Table S1, available in the online Supplementary Material). Our highly targeted method generated two results which we deemed false-positive identifications, with two cases displaying results consistent with the *B. anthracis* ‘Ames Ancestor’ strain used as a reference in the highly targeted assembly. In contrast, the assembly in our targeted method for those samples produced a genome which could not be assigned a *B. anthracis* sublineage or cgMLST, and our untargeted and semi-targeted methods for those samples reconstructed *B. cereus* genomes. KrakenUniq confirmed that for those samples, *B. cereus*, not *B. anthracis*, was highly abundant. Across all samples, cgMLST and CanSNPer results were always consistent within a sample.

**Table 2.** Description of the four bioinformatic methods used

	UntargetedMethod 1	Semi-targetedMethod 2	TargetedMethod 3	Highly targetedMethod 4
Genomic content targeting	After assembly	After assembly	Before assembly	Before assembly
Genomic content targeting method	MetaWrap -CONCOCT, metabat2, MaxBin2	BV-BRC metagenomic binning	Bowtie2 read recruitment	Bowtie2 read recruitment
Assembly method	MEGAHIT	MEGAHIT	SPAdes	SPAdes reference assembly
Taxonomic assignment	GTDBtk	GTDBtk	GTDBtk	GTDBtk
CanSNP assignment	CanSNPerBacillus_anthracis	CanSNPerBacillus_anthracis	CanSNPerBacillus_anthracis	CanSNPerBacillus_anthracis
cgMLST assignment	PubMLST*B. anthracis* cgMLST	PubMLST*B. anthracis* cgMLST	PubMLST*B. anthracis* cgMLST	PubMLST*B. anthracis* cgMLST
Computational cost	High	Medium	Low	Low

In ten cases where isolates had been obtained, genomes were generated from both isolates, as well as the complex samples from which isolates were obtained. Of these ten cases, seven complex samples produced reconstructed *B. anthracis* genomes with sublineage assignments that matched the sublineage assignment of the isolate genome. For the three cases that did not match, all were cases in which A.Br.047 was assigned to the reconstructed *B. anthracis* from the complex sample, whilst A.Br.014 was assigned to the isolate *B. anthracis*. In two of the three cases, A.Br.047 was also present in other samples, including other isolates, in the same outbreak. This may suggest that the disagreement between isolate and complex sample assignments could be due to multiple sublineages present in a sample. In one case where A.Br.047 was not otherwise detected, we sequenced a second extract of the complex sample and recovered an A.Br.047 assignment the second time.

**Fig. 1.**
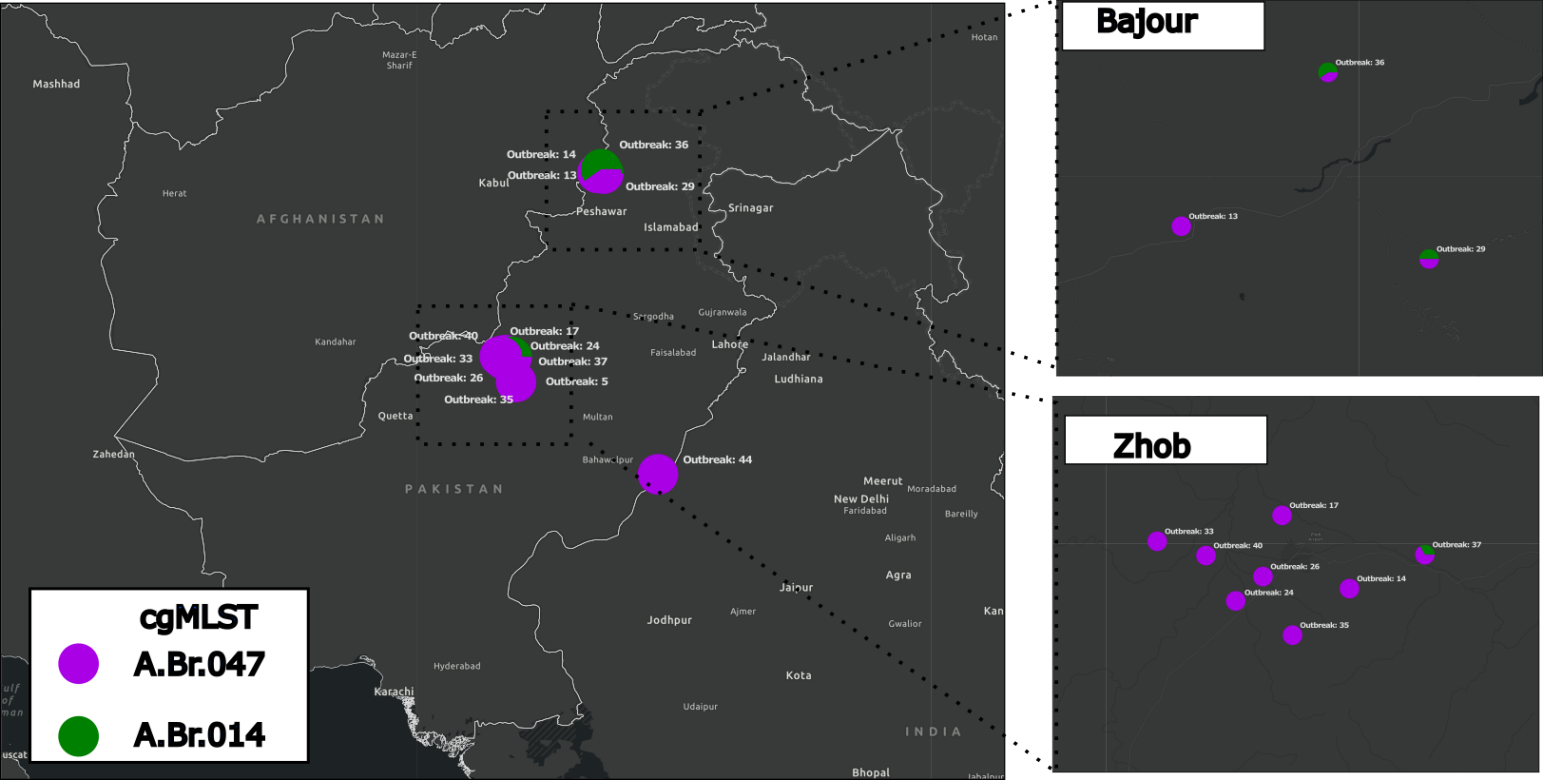
GIS Map of outbreaks in Pakistan, with pie charts coloured by proportion of cgMLST assignments for samples in that outbreak. Bajour (the northernmost region plotted on the whole map) and Zhob (the central region plotted on the whole map) are districts in Pakistan.

Samples were collected across 13 outbreaks in Zhob, Bajour and Fort Abbas in Pakistan. Each outbreak contained a single dead animal, and each sample came from blood-stained soil surrounding that animal. Seventeen samples were collected from soil surrounding sheep, three from goats and eleven from cows. We assigned a sublineage to at least one sample across all 13 outbreaks. An outbreak was defined as a single identified animal, and as a result, all samples from an outbreak came from blood-stained soil near a single animal carcass. Sublineage A.Br.047 Vollum was by far the most dominant sublineage and was present in all 13 outbreaks (Fig. 1, Table 3). Sublineage A.Br.014 Aust94 was found in two of three outbreaks sampled in Bajour but only one of nine outbreaks in Zhob.

**Table 3.** Samples from Pakistan summarized by outbreak. Percentage of each outbreak assigned to A.Br.014 and A.Br.047, along with an absolute number of samples with those assignments in parentheses. Included in these counts are 39 samples in total: 10 isolate sample assignments plus 29 metagenomic sample assignments. Outbreak numbers were previously assigned and correspond to those in Fig. 1 and Table S1

A.Br.014	A.Br.047	Outbreak	Province	Animal	Date
0	100% (5)	5 (2019)	Zhob	Goat	Apr-19
0	100% (1)	44 (2021)	Fort Abbas	Cow	Mar-21
0	100% (1)	40 (2020)	Zhob	Sheep	Jul-20
33.3% (2)	66.6% (4)	37 (2020)	Zhob	Sheep	Jun-20
60% (3)	40% (2)	36 (2019)	Bajour	Cow	Oct-19
0	100% (1)	35 (2019)	Zhob	Sheep	Oct-19
0	100% (1)	33 (2019)	Zhob	Sheep	Sep-19
50% (2)	50% (2)	29 (2019)	Bajour	Cow	Sep-19
0	100% (5)	26 (2019)	Zhob	Sheep	Aug-19
0	100% (3)	24 (2019)	Zhob	Sheep	Aug-19
0	100% (1)	17 (2019)	Zhob	Sheep	Aug-19
0	100% (4)	14 (2019)	Zhob	Cow	Jun-19
0	100% (2)	13 (2019)	Bajour	Cow	Jul-19

## Discussion

Our results suggest that computational reconstruction of *B. anthracis* can be employed in the absence of the ability to isolate *B. anthracis* and used to assign sublineage through cgMLST. Due in part to high clonality, the assembly of *B. anthracis* in complex samples led to reconstructed genomes of sufficient contiguity and with sufficient cgMLST loci to be considered adequate for cgMLST typing [7]. This does not require targeted enrichment of *B. anthracis* and can operate when *B. anthracis* represents at or above 1.65% of the total DNA, which is the lowest percentage of *B. anthracis* DNA found in a sample from which a *B. anthracis* genome could be reconstructed. Due to the extremely high load of *B. anthracis* in the bloodstream of infected animals [16], when collecting samples that are mixtures of blood and soil, the percentage of the sample that is *B. anthracis* is on average nearly 50%. However, the manner in which our data were collected means that we cannot meaningfully identify the limit of detection of this method, and more careful laboratory experimentation will be necessary in this regard.

When comparing bioinformatic methods, a targeted approach (method 3) appeared to be optimal, as it produced the highest number of reconstructed genomes without any false positives. The use of reference-based assembly in the highly targeted approach (method 4) resulted in false positives where sufficient *B. anthracis* sequences were not present. This is likely due to the reference-based assembly algorithm inserting reference genome DNA sequences in gaps when no sequences are provided by the short reads themselves. Consistency in cgMLST assignments within a sample was compelling, especially as assembly methods varied between MEGAHIT [23] in untargeted and semi-targeted and SPAdes [21] in targeted and highly targeted methods. Whilst complex sample assignments were not always consistent with isolate genome assignments, it is likely that this is due to multiple lineages of *B. anthracis* within a single animal.

Computational resources and operational usability also favoured a targeted approach (method 3). The untargeted approach (method 1) required roughly 10 min to assemble a sample, between 1 and 2 h for untargeted binning and bin refinement and 1 h for taxonomic assignment of bins. This resulted in at least slightly more than 2 h of run time per sample. The semi-targeted approach (method 2) could be carried out using fewer in-house resources at ~20 min per sample, as binning occurs using BV-BRC resources which can be accessed with a free-use account [30]. However, this does require an internet connection with the capacity to upload each assembly file, which may range from 5 to 500 Mb. A targeted approach (method 3), meanwhile, requires a read mapping step with a duration of ~5 min per sample and an assembly step with a duration of ~10 min per sample, resulting in less than 20 min per sample overall, without a need for internet connectivity. And whilst this study was carried out using a well-resourced compute node, all of the software discussed is open source and could be run on a typical Linux workstation, by an individual with nominal command line and Linux familiarity.

We identified multiple cases in which both A.Br.047 Vollum and A.Br.014 Aust94 were identified in samples from the same animal. As this occurred in two isolate genomes from outbreak 37 in Zhob, this cannot be attributed to computational methods. We also saw this in outbreak 36, where both isolate genomes were A.Br.014 Aust94 and the accompanying complex samples were A.Br.047 Vollum and A.Br.014 Aust94, respectively. This suggests that multiple distinct infection events could have occurred in these animals, with different sublineages in each event. Given the high seroprevalence of antibodies against *B. anthracis* in otherwise healthy, unvaccinated animals in Pakistan [3], repeated infections with *B. anthracis* may not be uncommon. Multi-sublineage infections could also be the result of an initial infectious dose of *B. anthracis* which itself contains multiple sublineages, as has been suggested previously [35,36]. Multi-sublineage infections may be detectable in a single complex sample, given future methodological improvement. Long-read sequencing is likely superior to short-read sequencing for the detection of these cases, given the ability to physically associate SNPs across longer reads. However, higher input DNA requirements for long-read sequencing are challenging, considering the harsh treatment requirements necessary for US HHS verification of no viable *B. anthracis* spores. However, long-read sequencing using an Oxford Nanopore MinION within Pakistan is a viable alternative. Future bioinformatic method development for Illumina short-read sequencing could lead to the ability to detect that a single sample contains multiple lineages, but the ability to identify which lineages are present would be limited. Otherwise, using current Illumina short-read sequencing methods, the acquisition of several independent samples from around the same carcass would be recommended to identify a multi-sublineage outbreak. Isolate-based assessment of sublineages using only a single sample per outbreak – using either whole-genome sequencing or traditional CanSNP analysis – would be unable to detect multi-sublineage outbreaks, even with methodological improvement, given the nature of enrichment necessary to generate isolates.

Our findings that A.Br.047 Vollum is the dominant sublineage in Pakistan are consistent with past surveys [1,7, 37], as the majority of *B. anthracis* genomes isolated from Pakistan have been identified as A.Br.047 Vollum. Whilst A.Br.014 Aust94 has been identified in neighbouring India [1,37], it has not been identified previously in Pakistan. As none of the provinces in which A.Br.014 Aust94 was detected bordered India, it is likely that A.Br.014 Aust94 is endemic throughout Pakistan but has not been detected due to a lack of sampling, rather than recent migration from India. Overall, sparse sampling in the region of *B. anthracis* makes comparison difficult. Indeed, whilst the closest reference genome to our A.Br.014 Aust94 genomes, A0656, was sampled in China, this sample was collected in 1982. Whilst PubMLST is a robust resource and contains 761 *B. anthracis* strains, more than 40% of those strains are from the USA or France, and only five are from Pakistan, all isolated before 2000. More frequent sampling, especially leveraging whole-genome sequencing, would greatly improve our understanding of the dynamics of the spread of *B. anthracis* in this region.

This study demonstrates that shotgun sequencing of complex samples is a viable method for identifying *B. anthracis* sublineage in the pursuit of outbreak tracking. We do not assess how quickly a deceased animal needs to be sampled after death, the ratio of blood to soil needed for recovery of *B. anthracis* or other parameters which will determine the overall usefulness of this method. However, this study represents a typical sampling campaign with difficult-to-access samples often in remote locations, without cold chain and with sequencing performed out of the country. As a result, our success suggests the general utility of this method.

## Supplementary material

10.1099/mgen.0.001422 Uncited Supplementary Material 1.

## Abbreviations

BV-BRC: Bacterial and Viral Bioinformatics Resource Center
cgMLST: core genome MLST
HHS: Health and Human Services
